# The Plastic Larval Body Color of the Pale Grass Blue Butterfly *Zizeeria maha* (Lepidoptera: Lycaenidae) in Response to the Host Plant Color: The Maternal Effect on Crypsis

**DOI:** 10.3390/insects14020202

**Published:** 2023-02-17

**Authors:** Ai Yoshida, Shintaro Yabu, Joji M. Otaki

**Affiliations:** The BCPH Unit of Molecular Physiology, Department of Chemistry, Biology and Marine Science, Faculty of Science, University of the Ryukyus, Okinawa 903-0213, Japan

**Keywords:** body color polyphenism, lycaenid butterfly, maternal effect, oviposition preference, *Oxalis corniculata*, phenotypic plasticity, transgenerational effect, *Zizeeria maha*

## Abstract

**Simple Summary:**

The larvae of many butterflies and moths show various body colors within a species. We studied the possible effect of the host plant color on the plastic larval body color of the lycaenid butterfly *Zizeeria maha*, which shows various larval body colors ranging from green to red, even within a sibling group. The females laid eggs normally on both green and red leaves, despite showing a green preference. The larvae grew equally by consuming either green or red leaves. The number of red larvae decreased as the larvae grew, demonstrating a stage-dependent variation. When the larvae were fed either green or red leaves across multiple generations, the red larvae were significantly more abundant in the red leaf lineage than in the green leaf lineage. Moreover, the red-fed siblings showed a significantly higher red larval frequency than the green-fed siblings in the red leaf lineage but not in the green leaf lineage. These results suggest that, in this butterfly species, the plastic larval body color for crypsis may be affected not only by the color of the leaves that the larvae consume but also by the color of the leaves that their mothers consume, in addition to a stage-dependent color variation.

**Abstract:**

Many lepidopteran larvae show body color polyphenism, and their colors may be cryptic on the host plant leaves. To elucidate the effect of the host plant color on the plastic larval body color, we focused on the lycaenid butterfly *Zizeeria maha*, which shows various larval body colors ranging from green to red, even within a sibling group. We showed that oviposition was normally performed on both green and red leaves, despite a green preference and the fact that the larvae grew equally by consuming either green or red leaves. The number of red larvae decreased from the second instar stage to the fourth instar stage, demonstrating a stage-dependent variation. When the larvae were fed either green or red leaves across multiple generations of the lineages, the red larvae were significantly more abundant in the red leaf lineage than in the green leaf lineage. Moreover, the red-fed siblings showed a significantly higher red larval frequency than the green-fed siblings in the red-leaf lineage but not in the green-leaf lineage. These results suggest that, in this butterfly species, the plastic larval body color for crypsis may be affected not only by the color of the leaves that the larvae consume (single-generation effect) but also by the color of the leaves that their mothers consume (maternal effect), in addition to a stage-dependent color variation.

## 1. Introduction

The high level of diversity in the body colors of animals, especially insects, has attracted the interest of many amateur and professional biologists alike. From the perspective of evolutionary biology, color polyphenism (polymorphism) is an important subject of research, because the coexistence of alternative body colors may indicate an intricate balance between different selection pressures that promote sympatric speciation [1,2,3,4,5]. Among the animals with diverse body colors, lepidopterans provide attractive systems that can be used to study ecological, evolutionary, and developmental mechanisms due to the extreme diversity in the wing color patterns of the adult butterflies [6]. Interestingly, lepidopterans show diverse colors not only on their adult wings but also on their larval bodies.

Several genes for larval color have been identified in lepidopterans, such as the silkworm moth *Bombyx mori* [7,8,9,10,11,12,13,14,15,16] and swallowtail butterflies [17,18,19]. In the Asian swallowtail butterfly *Papilio xuthus*, the juvenile hormone is responsible for the drastic change in the larval color patterns from the fourth instar to fifth instar stages [20]. In addition, melanin synthesis is under ecdysteroid control in the larvae of *P. xuthus* [21] and the tobacco hornworm moth *Manduca sexta* [22,23]. In these cases, despite hormonal controls, the larval body color is genetically determined without being affected by environmental factors. On the other hand, the larval body color is often plastic in lepidopterans, including swallowtail butterflies. For example, in the black swallowtail *Papilio polyxenes*, the larval body color at the last instar stage is seasonally determined [24]. In the pipevine swallowtail *Battus philenor*, the larval body color is determined in response to temperature, which is likely an adaptation to high-temperature environments [25]. Therefore, the contributions of environmental factors to the lepidopteran larval body color appear to be important, in addition to genetic factors.

In addition to swallowtail butterflies, the polyphagous larvae of the cotton bollworm moth *Helicoverpa armigera* show body color polyphenism which is dependent on the diet (i.e., host plants and parts of a host plant) [26,27]. Similar polyphenisms that are sensitive to environmental factors have been reported in some species, including the velvetbean caterpillar *Anticarsia gemmatalis* [28,29], the poplar and eyed hawkmoths *Lathoe poluli* and *Smerinthus ocellata* [30], and the common cutworm *Spodoptera litura* [31]. Generally, it can be understood that these colors depend on the color of the leaves on which the larvae rest, functioning as cryptic colors.

Many lycaenid species of the subfamily Polyommatinae and the tribe Polyommatini in Japan show larval body color variation ranging from green to red [32,33,34]. For example, such color variation can be observed in the grass blue butterflies *Zizeeria* and *Zizina*. However, understanding this larval color variation in *Zizeeria* and *Zizina* in terms of crypsis is not straightforward. The variation is observed even when identical diets are offered to siblings [35,36], suggesting that subtle genetic or environmental differences within a sibling group may contribute to such color variation. This larval body color may fall under genetic control, at least partially, and due to this variation, the body color of a given larva may not necessarily fit the leaf color on which that larva rests. In this case, the body color may often be counter-cryptic at the individual level. At the population level, due to host plant color variation, the body color variation may still be functional in terms of crypsis. There may be a mechanism for matching the body color with the host plant leaf color in response to environmental cues, without this process being affected by genetic variation. Considering these points, the biological functions of larval body colors and their determination mechanisms in lycaenid butterflies are of special interest.

Fukuda (2014) [37] noted that the larval body color of the cycad blue butterfly *Chilades pandava* (Lycaenidae) is basically reddish, a color which is potentially aposematic, reflecting larval toxicity, but green larvae are also found on green leaves, which may enhance crypsis. In the common hedge blue butterfly *Acytolepis puspa* (Lycaenidae), which has green and red larvae together with intermediate ones, the larvae eat the green and red leaves of the host plant, and the red color becomes evident at the third instar stage [38]. The same study [38] also briefly mentioned, without experimental data, that the larval body color of *A. puspa* is not related to the leaf color and further presented data that support the idea that the body color is affected by day length and temperature. Although the degree of the body color depends on the factors that are likely to vary depending on the species, it seems that lycaenid larvae make use of certain environmental cues to determine their body color, which probably has adaptive value. The lycaenid larval body color is thus considered as an expression of phenotypic plasticity, as in other biological systems [39,40]. The plastic phenotype is often determined in direct response to environmental factors, but the factors experienced by a previous generation (mother or father) may generate a parental (maternal or paternal) effect [41,42,43,44].

According to Uller (2008) [43], phenotypic plasticity via maternal effects (maternally mediated plasticity) may evolve under the following three conditions: (1) the environmental conditions fluctuate across generations, (2) the environmental conditions of the offspring are predictable based on those of the parents, and (3) the procurement and transmission of environmental information and its response are not costly. Considering that these conditions may be met in the case of lycaenid butterflies that do not disperse themselves over long distances [45], maternal effects may moderately affect the lycaenid larval body color. Maternal effects should be experimentally distinguished from single-generation (within-generation) effects, in which environmental factors directly act on the larvae within a single generation.

Maternal effects are often associated with life history traits in insects. Accumulated evidence suggests that robust mothers produce robust offspring [41,42]. In this context, nutritional requirements regarding the diets of mothers may influence the fitness and other plastic traits of their offspring. Moreover, polyphenism is often mediated by maternal effects in aphids, beetles, locusts, and other insects, which are likely adaptive [41,42]. However, to our knowledge, the maternal effects of the host plant on the butterfly larval body color polyphenism have not been well studied.

**Figure 1:**
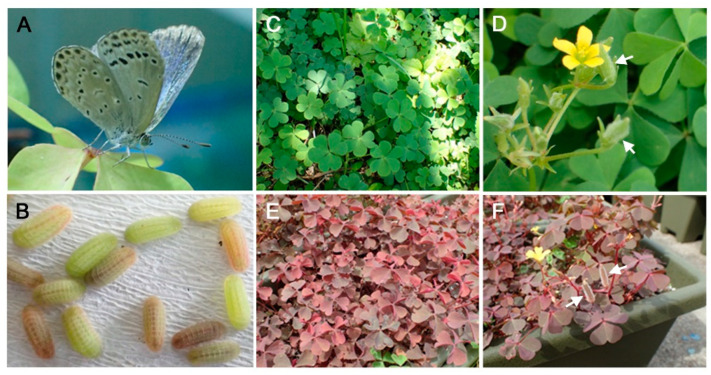
Butterfly and its host plant examined in this study. (**A**) An adult butterfly. (**B**) Larvae with various degrees of green or red body colors. (**C**,**D**) The green form of the host plant. Arrows indicate green fruits. (**E**,**F**) The red form of the host plant. Arrows indicate red fruits.

In this study, we focused on the larval body colors of the pale grass blue butterfly *Zizeeria maha*, which is a small lycaenid butterfly distributed in Japan [32,33,34] (Figure 1A). This butterfly has been used as a versatile model organism to bridge various biological phenomena in the field and laboratory, including the genetic assimilation of plastic phenotypes in the field [46,47,48], transgenerational effects, adaptive evolution, the field–laboratory paradox in radioactively contaminated areas in Fukushima [49,50,51,52], and the developmental mechanisms of wing color patterns [53,54,55]. Larvae of this species show various body colors, ranging from green to red (Figure 1B). Interestingly, the host plant of this butterfly species, the creeping wood sorrel *Oxalis corniculata*, also has two forms, the green form (Figure 1C,D) and the red form (Figure 1E,F), together with their intermediate forms observed in the field. Because larval and leaf color variations coincide, and because the red larval color of this species is not as vivid as the potential aposematic larval body color of *C. pandava*, the larval body color of *Z. maha* most likely functions as plastic crypsis.

In the present study, we first examined whether both the green and red forms of *O. corniculata* are suitable for oviposition and larval diets. We then described how larval body colors change as the larvae grow. On the basis of these findings, we examined whether the larval body color of this species is affected by an environmental factor, i.e., the color of the host plant leaves. More precisely, we hypothesized that the larval body color is affected not only by the color of the leaves that the larvae themselves consume (single-generation effect) but also by the color of the leaves that their mothers have previously consumed as the larvae (maternal effect). To this end, we reared three lines (Lines C, D, and E) on the red leaf diet (collectively called the red leaf lineage) and another three lines (Lines F, G, and H) on the green leaf diet (collectively called the green leaf lineage). These six lines were accompanied by branching siblings with different leaf color diets. We quantitatively compared the red larval frequencies between these lines, siblings, and generations and discussed the possible contributions of a single-generation effect and a maternal effect to the larval body color of this species.

## 2. Materials and Methods

### 2.1. Butterfly and Its Host Plant

The pale grass blue butterfly *Z. maha* (Figure 1A) and its host plant, the creeping wood sorrel *O. corniculata* (green form) (Figure 1C,D), were collected from the Nishihara campus of the University of the Ryukyus and its vicinity. This butterfly species is distributed throughout Japan, except for Hokkaido [32,33,34,56], and is the most abundant butterfly found in Japan [57]. The host plant has green and red forms, and the latter is called *O. corniculata* f. *rubrifolia* [58]. The less reddish forms are often called *O. corniculata* f. *tropaeoloides* [58] and *O. corniculata* f. *atropurpurea* [59]. In the present study, we used plants with leaves, stems, calyxes, and fruits that were dark red as the red form of this species. In addition to a collection from the university campus, red form plants were collected from mainland Japan (Tokyo and Kyoto) and sent to the university for experimental use (Figure 1E,F).

### 2.2. Plant Cultivation

Several planters (L540 mm × W190 mm × H200 mm) were each filled with 6 kg of commercially available cultivation soil, Hanasaki Monogatari (Akimoto Tensanbutsu, Iga, Mie, Japan). Several individuals of the wild plants (green and red forms of *O. corniculata*) were planted and cultivated under direct sunlight (Figure 1C–F). This plant species is known as a weed that propagates rapidly and is difficult to eradicate in the field. This plant propagates by “creeping” on the surface of the ground. We watered the plants every day and observed such a propagation in the planters. Later, to further facilitate propagation, numerous seeds were obtained and planted in several planters. The planting and seeding processes were repeated. These plants were used for the experiments. Cultivation without direct sunlight caused the leaf shoots of the red leaf plants to become slightly greenish, but this leaf color change required days or weeks to emerge. Thus, for larval feeding, leaves were collected by hand from the planters set under direct sunlight immediately before use. In this way, the red or green leaves kept their original colors when eaten by the larvae. The leaves were obtained with relatively long stems, and they were bundled together to be placed in a container for the larvae.

### 2.3. Butterfly Rearing

The egg collection and larval rearing procedures were performed in reference to previous studies [36,60] with minor modifications. Wild-caught adult butterflies (three females and two males) were confined in a glass container (L300 mm × W300 mm × H300 mm) together with a pot of the host plant (green or red form), flowers of *Bidens pilosa* and *Wedelia trilobata* collected from the university campus, and a Petri dish containing artificial nectar (POCARI SWEAT, Otsuka Pharmaceutical, Tokyo, Japan) under 18L:6D light conditions at 24 °C. This egg collection process started between 12:00 noon and 1:00 p.m. Several days later, the whole plant, with many eggs on its leaves, was isolated and left to stand until the eggs had hatched and the larvae had made unique food marks on leaves. The larvae were then transferred to a transparent plastic container (L140 mm × W140 mm × H55 mm) and reared on either green or red leaves of *O. corniculata* until they became pupae so as to record larval body color (see below). The pupae were collected individually in Petri dishes, placed vertically, and left quietly until the time of eclosion.

For the successive crosses of the rearing lines, females were selected from the group of individuals that eclosed at the peak eclosion days, that were relatively large in body size, and that appeared healthy in morphology and behavior. Their larval body colors were not specified. Three females and two wild-caught males (but see below for the behavioral tests) were confined in a glass container, as described above, to collect eggs at the beginning of every generation. The introduction of wild-caught males was considered necessary to ensure that the lines of the successive crosses would be robust enough to produce the next generation with minimal mortality and abnormality rates [53]. Because the red form of the plant is rare in the field, especially in Okinawa, the wild males caught in Okinawa likely consumed the green or intermediate form of the plant at the larval stage, in which case a potential paternal effect, if any, would be evenly applied to all the lines. The genetic background was not controlled among these males, but they were collected from a similar site so as to minimize genetic differences.

To minimize the possible contributions of genetic effects to the larval body colors, we primarily compared the collective results of the red leaf lineage and the green leaf lineage (see below). Furthermore, a sibling group (which was considered genetically homogeneous enough for physiological comparisons) was randomly divided into two subgroups, one of which was fed red leaves and the other green leaves. These two subgroups of the same sibling group were compared in terms of the red larval frequency (see below).

### 2.4. Behavioral Tests

For all the behavioral tests, adult butterflies were confined in a glass container under the conditions mentioned in Section 2.3. To quantitatively examine whether the female butterflies can naturally oviposit on the red form plant in addition to the green form plant, we first performed a sequential choice behavioral test. For this test, adult butterflies (three females and one male) were confined in a glass container, as mentioned above, and we first presented the butterflies with a green form plant (Plant B1) for 24 h. The next day, after confirming that the females were acclimated in the new environment and showed natural behaviors, including oviposition on a green form plant, we replaced the green form plant with a red form plant (Plant A) and left it for 48 h. The next day, the two females and one male in the container were replaced with two new females and one new male. These new individuals started with the red form plant. We confirmed that these females showed natural behaviors similar to those of the previous females. The next day, we again changed the red form plant (Plant A) to a green form plant (Plant B2) and left it for 24 h to verify whether females could continue oviposition. In this way, one set of females first experienced the green form plant for one day and then experienced the red form plant for one day, and the other set of females first experienced the red form plant for one day and then experienced the green form plant for one day.

The plants for oviposition were collected from the university campus and its vicinity. Plant replacement and termination were executed at 12:30 p.m. After termination, the number of eggs on the plants was counted, which was considered as the number of oviposition behaviors. Due to the procedure above, the results included eggs from the females who experienced the order of the green to red form and eggs from the females who experienced the order of the red to green form, averaging a possible effect of the presentation sequence. This sequential presentation was designed to test the capability of the females to oviposit on the red form plant when no alternative plant form was simultaneously available. In this sense, it was expected that the females would either stop oviposition or perform oviposition on the red form plant as a compromise. Alternatively, the females may oviposit on the red form plant as frequently as the green form plant. The number of eggs obtained in this sequential presentation was subjected to the *χ*^2^ goodness-of-fit test under the null hypothesis that the female butterflies would not differentiate between the green form and red form plants for oviposition.

After the sequential presentation, the eggs on the green form and red form plants were collected, and the hatched larvae were reared, as described above, to the adult stage. To examine whether the diet was suitable for larval growth, the survival rate (the number of eclosions divided by the number of larvae) and the pupal eclosion rate (the number of eclosions divided by the number of pupae) were obtained. The numbers of dead and surviving individuals were compared between the green form and red form plants using Fisher’s exact test.

We then performed a two-choice behavioral test to further understand the oviposition preference. For this behavioral test, three wild-caught females were confined in a glass container, as mentioned above, where the green form and red form plants were presented simultaneously for three days. The right and left positions of the presented plants were reversed on the second day. After the presentation period, the numbers of eggs on the plants were counted. In this test, the number of eggs represented the quantification of the number of oviposition behaviors. The entire procedure was repeated twice, and because multiple female individuals were used simultaneously for this test, the number of eggs obtained in these trials was summed for the *χ*^2^ goodness-of-fit test under the null hypothesis that the female butterflies would not differentiate between the green form and red form plants for oviposition.

### 2.5. Body Color Scores and Single-Individual Tracking

We took pictures of all the larvae to perform the red or green judgments and scored the cuticular color individually. Body color scores (0 to 3) were assigned to all the larvae according to the reddish portion and reddish darkness of the larval bodies based on visual inspection. Score 0 was defined as a pale yellow to yellow-green color, with no reddish portion. Score 1 was defined as a pale reddish color in the abdomen, especially at the caudal extremity. Score 2 was defined as a pale reddish color from the head to the abdomen throughout the body. Score 3 was defined as a dark reddish color throughout the body. Because of these somewhat complicated definitions, we did not use imaging software to score the larval body colors. Except for the F_0_ generation of Line C, which was judged only once, the scores were judged twice on different days to render the judgments more accurate and objective. Some larvae showed a dark background, but the judgment process was focused on the reddish portion. In the case of the small larvae, the color of the leaves consumed was occasionally observed through larval skin, but we focused on the cuticular skin color.

We used the body color scores for single-individual body color tracking. For the red leaf group, we selected 30 individuals (10 from Line D (F_1_), 7 from Line D (F_2_), and 13 from Line E (F_2_)). For the green leaf group, we selected 38 individuals from Line E (F_2_). They were individually confined in a plastic Petri dish and imaged at three time points with intervals of one, two, or three days. For simplicity, they were considered as larvae at the second instar, third instar, and fourth instar stages at these time points. Triplicate pictures were taken at each stage. Based on these pictures, scores were assigned to each larva at each stage by visual inspection.

### 2.6. Lineages and Lines

We established two “lineages” for comparison: the red leaf lineage (reared on red leaves across generations, from F_0_ to F_3_) and the green leaf lineage (reared on green leaves across generations, from F_0_ to F_3_). For each lineage, three independent genetic “lines” were produced from three founder females and two males in each case. To test for maternal effects, we constructed sibling subgroup branches by feeding leaves of the opposite color to a portion of the siblings. These two sibling subgroups were considered genetically indistinguishable. See the Results, Section 3.3, for the lineages, lines, and sibling subgroup branches that were produced in this study.

For simplicity, the red larval frequency was used to evaluate maternal and single-generation effects in successive crosses. The red larval frequency was defined as the number of red larvae (including those with scores of 1, 2, and 3) divided by the total number of larvae examined and was expressed as a percentage. For the evaluation, all individuals were pictured within a few days after counting the number of individuals (14 days after the beginning of egg collection). Scores were ascribed to all individuals based on digital photographs, as mentioned above. These were mostly second instar larvae. Because third instar and fourth instar larvae were rare at that point, and because scores 1, 2, and 3 were all considered to be a single category of red larvae, the data of all the individuals were used to obtain the red larval frequencies.

### 2.7. Definitions of Operational Effects

When examining each line in this study, we presumed that genetic contributions to the larval color could be ignored for the sake of simplicity and the analyses. This presumption may not necessarily be correct, but based on this presumption, we defined an “operational” maternal effect as a statistically significant change in the red larval frequency from the previous generation of a line based on Fisher’s exact test. Similarly, an “operational” single-generation (within-generation) effect was defined as a significant difference in the red larval frequency between the main line and a branching sibling subgroup that consumed leaves of different colors. The statistical significance was evaluated by the number of red larvae (scores 1, 2, and 3) and the number of green larvae (score 0) using Fisher’s exact test.

Theoretically, we expected the following model dynamics for the red larval frequencies if maternal effects functioned in the red leaf lineage: an increase in the red leaf frequency from the previous generation, followed by its stabilization in the next generation. This simple model considers increases in the red larval frequency on a generation-by-generation basis. At the same time, we expected that the red larval frequency of the sibling subgroup that consumed green leaves may be lower than that of the main red line. This difference assumes that green leaves directly affect the larval body color through a single-generation effect. Furthermore, this difference would be nullified in the next generation, which may be due to the suppression of the single-generation effect by a maternal effect. Thus, based on these model dynamics of the red larval frequencies, we expected a reinforcement of the maternal effects over generations. This model was also applied to the green leaf lineage.

### 2.8. Statistical Analysis

Statistical tests (*χ*^2^ goodness-of-fit test, Student’s *t*-test (two-tailed, either paired or unpaired), and Fisher’s exact test) were performed using Microsoft Excel (Office 365) and JSTAT 13.0 (Yokohama, Japan). The results were not adjusted by Bonferroni or other methods for simplicity. Statistical significance was judged using the conventional border, *p* < 0.05.

## 3. Results

### 3.1. Suitability of the Red Form Plant: Oviposition Preference and Larval Growth

**Figure 2:**
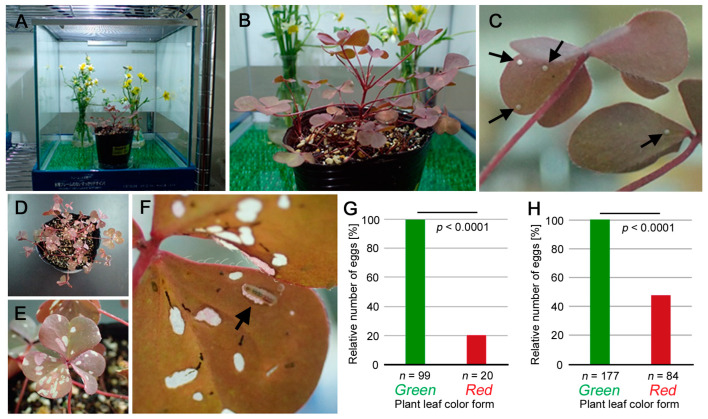
Suitability of red form plants for oviposition behavior and larval growth. (**A**) Presentation of the red form plant in a glass container. The green form plant was presented before and after the red form plant in the same set-up. (**B**) High magnification of (**A**). (**C**) Eggs deposited on the red form leaves (arrows). (**D**) Top-down view of the red form plant several days after the beginning of the egg collection process. (**E**) High magnification of (**D**). (**F**) A larva on the lower side of a red form leaf (arrow). (**G**) Sequential choice behavioral test for oviposition preference. The number of eggs was counted and expressed as relative percentages. The number of eggs on the green form plant was adjusted to 100%. The indicated *p*-value was a result of the *χ*^2^ goodness-of-fit test. (**H**) Two-choice behavioral test for oviposition preference. The number of eggs was counted and treated as in (**G**).

We first examined whether the red form of *O. corniculata* could support oviposition behavior in comparison to the green form using a sequential presentation protocol (see Section 2). After this sequential presentation (Figure 2A,B), we obtained 99 eggs on the two green form plants and 20 eggs on the red form plants (Figure 2C; Table S1). Larvae hatched from these eggs (Figure 2D–F) and grew to the adult stage on either red or green leaves. The survival rate to the adult stage (eclosion) was 95% for the red leaf group (*n* = 20) and 98% for the green leaf group (*n* = 99) (*p* = 0.43; Fisher’s exact test) (Table S1), demonstrating that both color forms were equally suitable as diets for larval growth. The pupal eclosion rate also showed similar values (Table S1). The ratio of eggs deposited on the green and red forms was approximately 5:1 for an equal presentation period (48 h). The numbers of deposited eggs were significantly different between the green and red forms (*p* < 0.0001; *χ*^2^ goodness-of-fit test) (Figure 2G).

To further understand the oviposition preference, we next performed a two-choice behavioral test by presenting the butterflies with both green form and red form plants simultaneously. We obtained 177 eggs on the green form plant and 84 eggs on the red form plant. The ratio was approximately 2:1, and the numbers of deposited eggs were significantly different between the green and red forms (*p* < 0.0001; *χ*^2^ goodness-of-fit test) (Figure 2H). Both the sequential and two-choice presentations indicated that the red form was suitable for oviposition, although it was less preferred by the females.

### 3.2. Single-Individual Tracking

**Figure 3:**
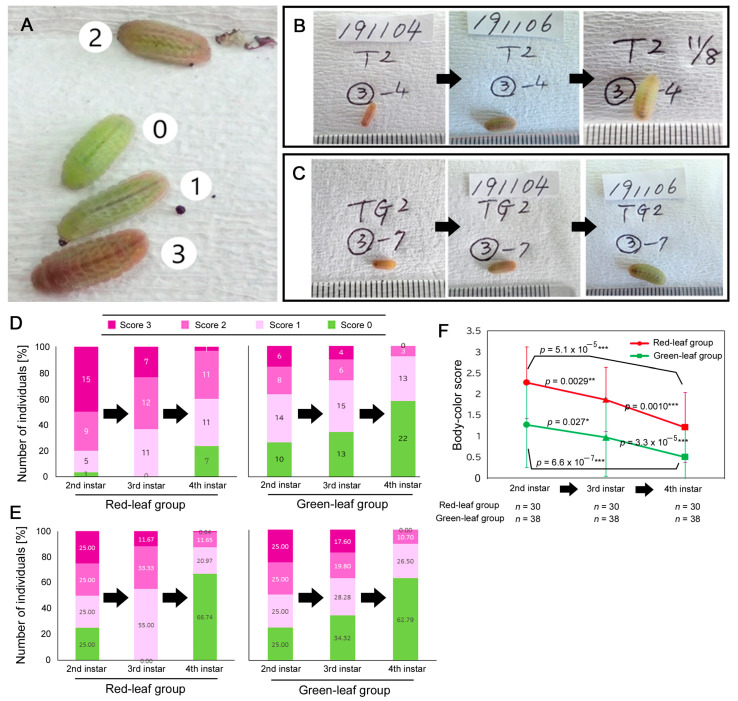
Changes in larval body colors over time. (**A**) Larvae with different body color scores. (**B**) A time-tracked individual with body color changes over time. Pictures were taken on 4, 6, and 8 November 2019. This larva belongs to Line E (F_2_), reared on red leaves. Scales indicate millimeters. (**C**) Another time-tracked individual. Pictures were taken on 1, 4, and 6 November 2019. This larva belongs to Line E (F_2_), reared on green leaves. (**D**,**E**) Number of individuals with different body color scores over time in the red leaf group (left) and the green leaf group (right). In (**E**), the starting percentages were adjusted to be equal (25% each) among larvae with different body color scores. (**F**) Changes in the body color scores in the red leaf group and the green leaf group over time. Asterisks indicate statistical significance: *, *p* < 0.05; **, *p* < 0.01; ***, *p* < 0.001 (bi-sided paired *t*-test).

According to the body color scoring system (Figure 3A) (see Section 2), we focused on 30 individuals from the red-leaf-eating group and 38 individuals from the green-leaf-eating group, which we reared individually in small Petri dishes. As shown in the examples (Figure 3B,C), the body color changed over time in both the red leaf and green leaf groups (Figure 3D–F, Tables S2–S5). The body color scores significantly decreased from the second instar stage to the third instar stage and from the third instar stage to the fourth instar stage in both the red leaf and green leaf groups (Figure 3F). Thus, red larvae were most frequent at the second instar stage and least frequent at the fourth instar stage, demonstrating a stage-specific effect.

### 3.3. Comparison between the Red Leaf and Green Leaf Lineages

**Figure 4:**
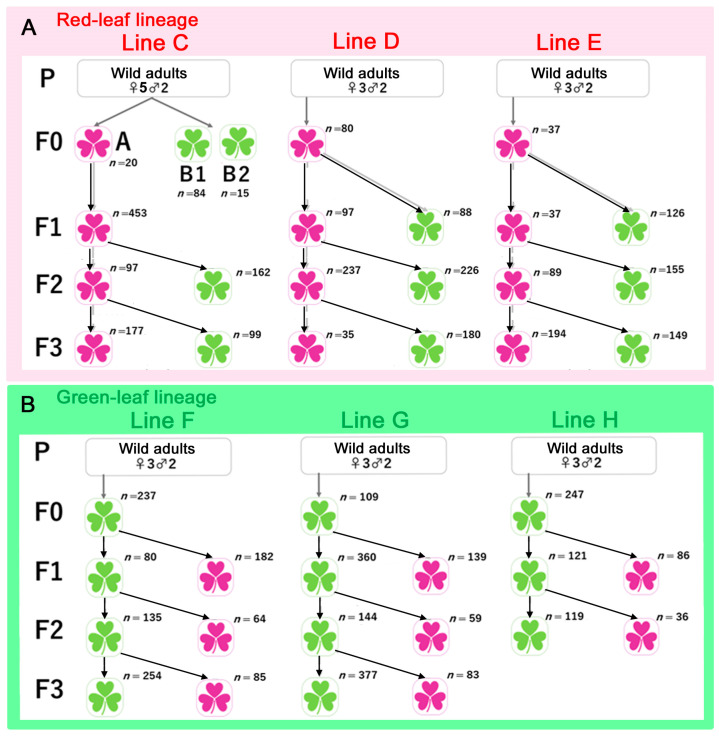
Experimental design of successive crosses with different leaf colors. The numbers of larvae examined in this study are shown. (**A**) Red leaf lineage (Lines C, D, and E). Wild adults were designated as the P generation. The next generation after the P generation was defined as the F_0_ generation, which consumed red leaves. Note the sibling subgroup branches that consumed green leaves from the main lines of almost every generation that consumed red leaves. In Line C, the red leaf group and the green leaf group in the F_0_ generation were designated as A and B, respectively. The designations of A, B1, and B2 relate to the sequential choice behavioral test. These individuals were not used for the subsequent statistical tests (Figure 5, Figure 6 and Figure 7). (**B**) Green leaf lineage (Lines F, G, and H). The arrangement of the green leaf lineage is basically a “mirror image” of the red leaf lineage.

**Figure 5:**
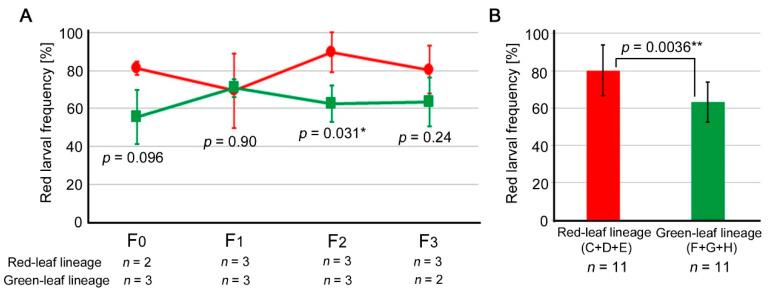
Changes in the red larval frequencies of the red leaf and green leaf lineages. Asterisks indicate statistical significance: *, *p* < 0.05; **, *p* < 0.01 (bi-sided unpaired Student’s *t*-test). (**A**) Changes over generations. The red larval frequencies were significantly different between the red leaf and green leaf lineages in the F_2_ generation. (**B**) The red larval frequencies of the red leaf and green leaf lineages across generations. A statistically significant difference was observed.

We summarized the “lineages” (red and green lineages) and “lines” (Lines C, D, and E for the red leaf lineage and Lines F, G, and H for the green leaf lineage) produced in this study (Figure 4, Tables S6–S10). When three lines were combined for each generation (excluding the sibling subgroup branches), the red larval frequencies were significantly different between the red leaf and green leaf lineages in the F_2_ generation (*p* = 0.031; Student’s *t*-test) (Figure 5A). The F_0_ and F_3_ generations did not show a statistically significant difference, despite the red leaf lineage having larger frequencies, but this could be due to the small sample size. Similarly, the fact that the F_1_ generation was without any difference could be due to the small sample size. When all the lines of all the generations were examined together, the red larval frequency of the red leaf lineage was significantly higher than that of the green leaf lineage (Figure 5B).

### 3.4. Comparison between the Red-Fed and Green-Fed Siblings

**Figure 6:**
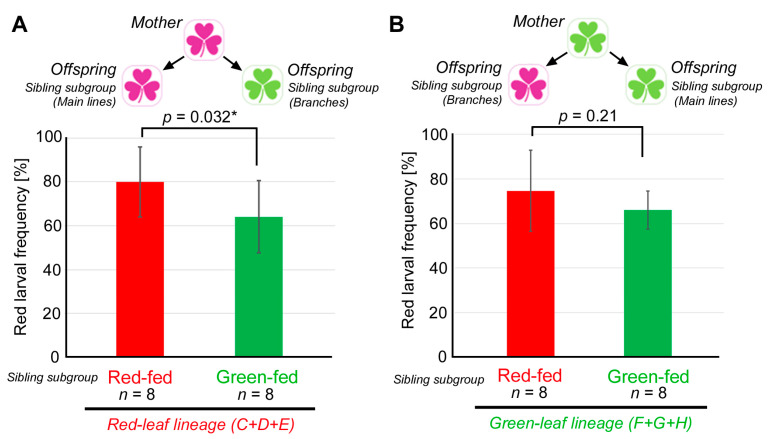
Sibling subgroup comparisons of the red larval frequency to test interactions between the maternal and offspring diets. An asterisk indicates statistical significance at the level of *p* < 0.05 (bi-sided paired *t*-test). (**A**) Red leaf lineage (Lines C, D, and E). (**B**) Green leaf lineage (Lines F, G, and H).

Although there appeared to be a lineage-dependent effect, as described in the previous section, we could not exclude the possibility that the genetic composition, instead of the leaf color consumption, contributed to such an effect. To circumvent this problem, a group of sibling individuals of each line in almost every generation were randomly divided into two sibling subgroups: a red-fed subgroup and a green-fed subgroup (Figure 4). They were considered genetically indistinguishable in the present analyses. When these sibling subgroups were compared in the red-leaf lineage (Lines C, D, and E), the red-fed sibling subgroups (the main line subgroups) showed significantly higher red larval frequencies than the green-fed sibling subgroups (the branching subgroups) (*p* = 0.032; paired Student’s *t*-test) (Figure 6A). Because these sibling subgroups were considered genetically indistinguishable, and because only the color of the leaves consumed by the present generation differed between these sibling subgroups, this result suggests a single-generation effect of the leaf color (instead of a genetic effect), contributing to this significant difference in the red larval frequency. However, this does not clarify the contribution of a maternal effect.

To examine whether a maternal effect may also have contributed to the results, the same comparison was performed on the green leaf lineage (Lines F, G, and H). In this case, the red-fed sibling subgroups (the branching subgroups) and the green-fed sibling subgroups (the main line subgroups) did not show a significant difference (*p* = 0.21; paired Student’s *t*-test) (Figure 6B). Regardless of the color of the leaves that the larvae consumed, the larval body color was not significantly affected. This result suggests that a single-generation effect did not strongly influence the green leaf lineage. Significantly, the only difference in the experimental conditions between the red lineage (Figure 6A) and the green lineage (Figure 6B) was the diet history of their mothers. Therefore, these results support the interpretation that a maternal effect, together with a single-generation effect, contributed to an increase in the red larval frequency in the red leaf lineage but not the green leaf lineage. Therefore, there appear to be both single-generation and maternal effects at work simultaneously, at least in the red leaf lineage.

### 3.5. Dynamics of the Individual Lines: Red Leaf Lineage

Here, we examine the dynamics of each line in terms of the red larval frequency based on the understanding that we could not exclude the possible contribution of the small sample size (i.e., experimental “genetic drift”) that might have been introduced in the case of each line. For the sake of argument, we defined the “operational” maternal effect and the “operational” single-generation effect for the subsequent analyses (see Section 2) and searched for these effects, although these operational effects do not necessarily indicate genuine maternal and single-generation effects.

**Figure 7:**
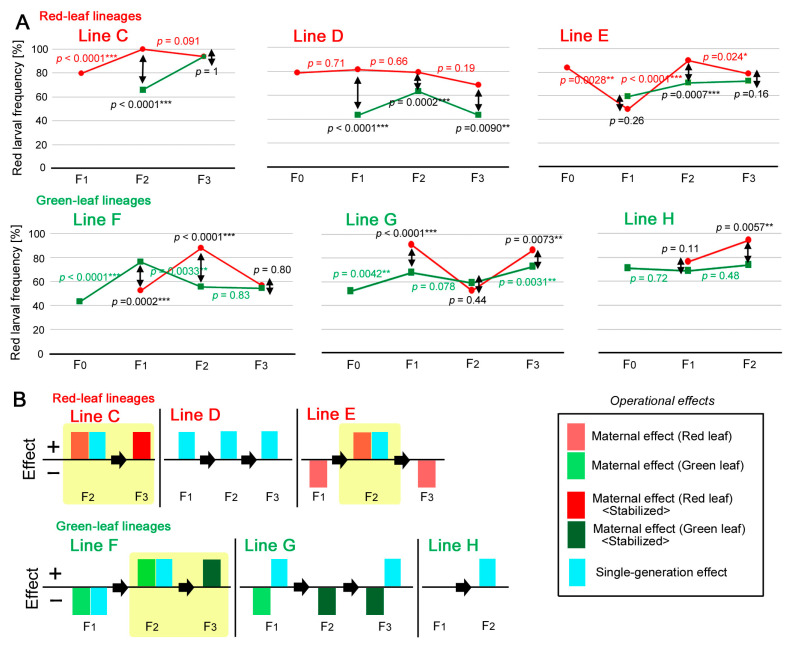
Operational maternal effects and operational single-generation effects in the six independent lines (Lines C, D, and E in the red leaf lineage and Lines F, G, and H in the green leaf lineage). For definitions, see the Section 2. Asterisks indicate statistical significance: *, *p* < 0.05; **, *p* < 0.01; ***, *p* < 0.001 (Fisher’s exact test). (**A**) Dynamics of the red larval frequencies. The upper three panels indicate the red leaf lineage, and the bottom three panels indicate the green leaf lineage. Red or green *p*-values indicate the results of the two successive generations. Black *p*-values indicate the results of a sibling subgroup branch and its corresponding main line (double-headed arrows) for a given generation. (**B**) Effect diagrams (interpretations of (**A**)) used to intuitively understand the operational maternal and single-generation effects. The upper three panels indicate the red leaf lineage, and the bottom three panels indicate the green leaf lineage. An effect is shown by a plus/minus unit box. Operational maternal effects are indicated by reddish boxes for the red leaf lines and by greenish boxes for the green leaf lines. Operational single-generation effects are indicated by blue boxes. A given generation has room for two boxes, one for the operational maternal effect and one for the operational single-generation effect, but no box is shown when no operational maternal or single-generation effect was detected. The experimental results that realized the theoretically expected model dynamics of the maternal effects are highlighted in yellow in Lines C, E, and F.

We first examined the red leaf lineage (Lines C, D, and E) (Figure 7A, top three panels). A simplified “effect diagram” was also drawn (Figure 7B, top three panels). In Line C, the level of the red larval frequency for the F_2_ generation was maximal (100%), and the difference between the F_1_ and F_2_ generations was statistically significant (*p* < 0.0001; Fisher’s exact test, in this case and hereafter), suggesting an operational maternal effect transferred from the F_1_ mothers to the F_2_ offspring. Simultaneously, a green leaf sibling subgroup in the F_2_ generation showed a significantly lower frequency than the main red leaf sibling larvae (*p* < 0.0001), suggesting an operational single-generation effect, possibly due to the consumption of green leaves. The high red larval frequency in the F_2_ generation was maintained in the F_3_ generation (*p* = 0.091). Importantly, a difference in the red larval frequency between the main red leaf line and the green leaf sibling subgroup was null in the F_3_ generation (*p* = 1), suggesting that the operational single-generation effect disappeared, and that the operational maternal effect may have stabilized in this generation. These results indicated that Line C showed a series of changes that were consistent with the theoretically expected model dynamics of the maternal and single-generation effects (see Section 2).

Line D showed red larval frequencies of approximately 80% or less across generations. No significant differences in the red larval frequency were detected between generations (*p* = 0.71 for the F_1_ generation, *p* = 0.66 for the F_2_ generation, and *p* = 0.19 for the F_3_ generation). The green leaf sibling subgroups always showed significantly lower levels than the main red leaf line across generations (*p* < 0.0001 for the F_1_ generation, *p* = 0.0002 for the F_2_ generation, and *p* = 0.0090 for the F_3_ generation). In other words, their differences were not nullified in any generation. These results indicated that in Line D, operational single-generation effects dominated, and operational maternal effects were not present.

In Line E, despite the red leaf consumption of the F_0_ and F_1_ generations, the F_1_ larvae showed a significantly lower red larval frequency (approximately 50%) than the F_0_ generation (*p* = 0.0028), which was operationally considered as a “negative” maternal effect. The sibling subgroup that consumed green leaves in the F_1_ generation showed a similar frequency (*p* = 0.26), suggesting that this generation was not responsive to the leaf color. In other words, whichever leaf color was consumed, red and green larvae were produced at an approximately 1:1 ratio. However, in the F_2_ generation, the red larval frequency suddenly increased significantly (*p* < 0.0001), suggesting an operational maternal effect on the red body color. In addition, in the F_2_ generation, the green leaf sibling subgroup showed a lower red larval frequency than the main red leaf line (*p* = 0.0007), suggesting a single-generation effect. Therefore, the dynamics of the F_2_ generation followed the theoretically expected model dynamics of maternal and single-generation effects. In the F_3_ generation, a significant decrease in the red larval frequency from the F_2_ generation was observed, despite the continuous consumption of red leaves (*p* = 0.024), again suggesting a negative operational maternal effect. Additionally, in this generation, both the main red leaf line and the green leaf sibling subgroup showed similar red larval frequencies (*p* = 0.16), suggesting that no operational single-generation effect was at work. In this line, it appeared that there were oscillating negative and positive operational maternal effects.

### 3.6. Dynamics of the Individual Lines: Green Leaf Lineage

Here, we examined the dynamics of individual lines in the green leaf lineage (Lines F, G, and H) (Figure 7A, bottom three panels). A simplified effect diagram was also drawn (Figure 7B, bottom three panels). In Line F, despite the consumption of green leaves, the red larval frequency increased from the F_0_ to F_1_ generation (*p* < 0.0001), suggesting a negative operational maternal effect. When red leaves were offered to a sibling subgroup in the F_1_ generation, the red larval frequency was lower than that of the main green leaf line (*p* = 0.0002), suggesting a negative operational single-generation effect. However, in the F_2_ generation, the red larval frequency of the green leaf line significantly decreased from the F_1_ generation (*p* = 0.0033), suggesting an operational maternal effect. The sibling subgroup that consumed red leaves showed a significantly higher red larval frequency than the main green leaf line (*p* < 0.0001), suggesting an operational single-generation effect. In the F_3_ generation, the red larval frequency was as low as that of the previous generation (*p* = 0.83), suggesting that the operational maternal effect was stabilized in the green leaf line. Furthermore, in the F_3_ generation, the sibling subgroup that consumed red leaves showed a red larval frequency similar to that of the main green leaf line (*p* = 0.80), suggesting that the operational single-generation effect was nullified. These results indicated that Line F showed a series of changes that were consistent with the theoretically expected model dynamics of maternal and single-generation effects (see Section 2).

In Line G, despite the consumption of green leaves, the red larval frequency increased in the F_1_ generation from the previous generation (*p* = 0.0042), suggesting a negative operational maternal effect. Simultaneously, the sibling subgroup that consumed red leaves showed a higher red larval frequency than the main green leaf line (*p* < 0.0001), suggesting an operational single-generation effect. The increase in the red larval frequency in the F_1_ generation was stabilized in the F_2_ generation (*p* = 0.078), and the red leaf sibling subgroup showed no difference from the main green leaf line (*p* = 0.44), suggesting that there was no operational single-generation effect. In the F_3_ generation, the red larval frequency increased again, despite the consumption of green leaves by the larvae (*p* = 0.0031), suggesting a negative operational maternal effect. The frequency of the sibling subgroup that consumed red leaves was again higher than that of the main green leaf line (*p* = 0.0073), suggesting that an operational single-generation effect reappeared.

In Line H, despite the consumption of green leaves, no decrease in the red larval frequency was detected across generations (*p* = 0.72 for the F_1_ generation and *p* = 0.48 for the F_2_ generation). In the F_1_ generation, the red larval frequencies of the main green leaf line and its corresponding red leaf sibling subgroup showed similar levels (*p* = 0.11), suggesting that the body colors were not affected by the leaf colors. However, in the F_2_ generation, the sibling subgroup that consumed red leaves showed a significantly higher red larval frequency than the main green leaf line (*p* = 0.0057), suggesting the existence of an operational single-generation effect.

## 4. Discussion

### 4.1. Oviposition Preference and Larval Growth

This study focused on the potential effect of leaf color on larval body color in the lycaenid butterfly *Z. maha*. At present, the functional significance of body color variation in this species is not completely clear, but the body color variation is likely cryptic, considering that the leaf and larval colors vary similarly from green to red (Figure 1). Although the frequency of the red form (used in this study) in the field is low in Japan, intermediate color forms are frequent. Moreover, color gradation can be observed in an individual plant, and in many individuals, the leaves and stems are somewhat reddish. These facts justify the possible cryptic function of larval body colors.

In this study, we first showed that the red form plant is suitable for oviposition in the case of *Z. maha* (Figure 2). We counted the number of eggs, which was used as a tool to quantify the oviposition behaviors as integers. The oviposition preference was higher for the green form plant in the sequential choice behavioral test (green/red = 5) and in the two-choice behavioral test (green/red = 2). Maturing eggs may have to be deposited within a certain period, because other maturing eggs are present behind. In the sequential presentation, females cannot stop ovipositioning and may oviposit on an unsuitable plant or object, resulting in a green/red ratio that is closer to one or to that of the two-choice test even if the red form plant is less preferred by females. Alternatively, the females may stop ovipositioning when only the red form plant is available, resulting in a green/red ratio that is much higher than one or than that of the two-choice test.

Under our experimental conditions for the sequential presentation, the green/red ratio was much higher than that of the two-choice test. This means that the females appeared to reduce the pace of oviposition when only the red form plant was available. We interpret that this oviposition behavior was not due to an unsuitable object, considering that the females deposited eggs on the red form plant even when the green form plant was available. The ratio was less biased (closer to one) in the two-choice test, which may suggest that females may oviposit on the red form plant more often when the green and red forms coexist at the same site. Under these circumstances, larvae may eat both green and red leaves, because they move from leaf to leaf as they grow. The green-over-red preference may reflect the fact that red leaves are darker than green leaves, and the red form plant is likely less visually recognizable by female butterflies. Moreover, lycaenid butterflies do not appear to have a photoreceptor for red wavelengths of light [61]. These results of the behavioral tests may reflect neurologically innate behaviors of this species.

Despite the skewed oviposition preference for green leaves, the larvae grew appropriately when consuming red leaves. Most likely, both green and red leaves contain sufficient levels of oxalic acid, a feeding stimulant for larvae [62]. There appears to be a discrepancy between the adult oviposition preference (biased) and larval performance (unbiased). Such a discrepancy is common in butterflies and other insects, such as *H. armigera* [63,64], *Asclepias* species [65], lycaenid *Mitoura* species [66], and lycaenid *Lycaeides* species [67,68]. This may be a general trend in herbivore host choice among insects [69], although oviposition preference and larval fitness coincide well in some species [70].

### 4.2. Decrease in Red Larval Frequencies during Larval Growth

By tracking the larvae individually, we showed that the red larval frequency decreased during larval growth regardless of the color of the leaves that the larvae consumed (Figure 3). That is, larval body color is a stage-dependent plastic trait in this species. It appears that in *Z. maha*, red pigment is produced more in the early stages. This result is different from that of another lycaenid butterfly, *A. puspa*, in which the red color becomes evident at the third instar stage [38]. In addition, the present result may suggest that red pigment precursors are deposited in eggs by mothers so that young larvae may readily synthesize the red pigment.

### 4.3. Lineage and Sibling Comparisons

Our experimental results for the single-generation and maternal effects were obtained from the lineages and lines shown in Figure 4. This experimental design is unique in that the main lines were accompanied by the branching sibling subgroups. Moreover, the green leaf lineages were designed to be a mirror image of the red leaf lineage. This design rendered examinations of the single-generation and maternal effects possible. However, we admit that it would be better to increase the number of lines and generations and to use similar numbers of individuals per generation. The use of various numbers of individuals per generation in the present study was due to the availability of the plant leaves and the availability of human power required to rear large number of larvae. These compromised numbers might have affected the statistical results to some extent.

Nevertheless, when the red larval frequency data of three lines in each generation were considered as an independent analytical unit, we observed a significant difference in the F_2_ generation between the red leaf and green leaf lineages (Figure 5A). Furthermore, assuming that each generation of any line is an independent analytical unit, a simple comparison between the frequency data of the red leaf and green leaf lineages showed a significant difference (Figure 5B). These results indicate that the red leaf lineage was redder than the green leaf lineage, suggesting some effects of the leaf color consumed by the larvae. In other words, these results suggest that the color of the host plant affects the larval body color and that either single-generation effects or maternal effects (or both) may play a role in expressing the larval body color.

We also compared two sibling subgroups, one of which were fed red leaves and the other green leaves (Figure 6). Because there was no genetic bias in this comparison, the significant difference between the red-fed and green-fed subgroups in the red leaf lineage (Figure 6A) suggests that a single-generation effect played a role in the increase in the red larval frequency in the red leaf lineage. Importantly, this single-generation effect appeared to be present only when mothers consumed red leaves, because a single-generation effect did not appear to be present in the green leaf lineage (Figure 6B). Between the red leaf lineage (Figure 6A) and the green leaf lineages (Figure 6B), there was only one difference in experimental conditions, i.e., the color of the leaves that the mothers consumed. Therefore, it appears that a maternal effect must be present to render a single-generation effect effective, and this nongenetic heritable (transgenerational) effect (i.e., maternal effect) is asymmetric in terms of the mothers’ leaf colors. It is likely that the green body color is the default for larvae of this species, and larvae take advantage of maternal information to potentially become cryptic on red leaves. Only under circumstances where the mother generation ate red leaves and where the present generation eats red leaves can an increase in the red larval frequency be realized. However, this is a general trend, and individual variation exists (Figure 7).

### 4.4. Dynamics of the Independent Lines

To analyze each line independently, we first clarified our operational definitions of the maternal and single-generation effects and presented our theoretically expected model dynamics of the maternal and single-generation effects over generations (see Section 2). With these definitions and models in mind, we observed that the larval responses varied in a line-dependent manner (Figure 7). In the red leaf lineage, Line C showed the theoretically expected model dynamics of the maternal effects in the F_2_ and F_3_ generations. Line D did not show any operational maternal effect but showed operational single-generation effects across generations. Line E showed negative and positive operational maternal effects but showed the theoretically expected model dynamics of the maternal and single-generation effects. All three lines showed different dynamics in terms of the red larval frequency, which may be attributed to genetic drift.

We nonetheless obtained three cases in which the theoretically expected model dynamics of maternal and single-generation effects were observed, where both operational maternal and single-generation effects were detected in the F_2_ generation. Among them, two cases showed that the operational maternal effect was stabilized in the F_3_ generation. Simultaneously, in the F_3_ generation, the operational single-generation effect was not present, which may be considered a result of suppression caused by the operational maternal effect. Importantly, these dynamics were detected not only in the red leaf lineage (Lines C and E) but also in the green leaf lineage (Line F). In the green leaf lineage, Line F showed the theoretically expected model dynamics of maternal effects, despite a negative effect in the F_1_ generation. Line G showed negative operational maternal effects together with single-generation effects. Line H showed an operational single-generation effect only once, without any operational maternal effect. The dynamics of Line F in the F_2_ and F_3_ generations were similar to those of the F_2_ and F_3_ generations of Line C with the color reversed.

Although these results do not exclude a possible contribution of genetic drift, operational maternal and single-generation effects likely affected the larval body colors in these lines. In contrast, operational single-generation effects were solely detected in Line D across generations. In both the red leaf and green leaf lineages, operational single-generation effects were detected in all the lines at least once per line, suggesting that operational single-generation effects also affected the larval body color in these lines. These “operational” effects do not necessarily indicate real maternal and single-generation effects. However, considering the results of the collective comparisons (Figure 5 and Figure 6), we believe that at least some of the operational effects were real effects in the case of the independent lines.

As discussed above, we carefully defined both operational maternal and single-generation effects. Somewhat surprisingly, based on these definitions, we detected “negative” operational maternal and single-generation effects. Negative effects are biologically difficult to interpret, because they appear to be counter-adaptive. An extreme interpretation of the negative effects is that the entire set of operational maternal and single-generation effects detected here were simply stochastic behaviors of the body color, without a direct response to the leaf color. However, the effect diagrams suggest that these effects do not appear to be stochastic. Rather, negative effects were only detected in specific lines. In concrete terms, Line E showed oscillating negative and positive operational maternal effects, and Line G showed consecutive negative operational maternal effects across generations. In this case, the negative operational maternal effects that first emerged in the F_1_ generation appeared to be stabilized in the F_2_ generation and enhanced in the F_3_ generation, suggesting that the expressiveness of the negative operational maternal effects was inherited in this line. That is, the red larval frequency increased in a generation-by-generation manner, despite the larvae’s consumption of green leaves, but their red-fed siblings were still redder. Therefore, it is likely that the negative effects, together with the line-dependent variation, may be attributed to genetic drift.

In summary, regardless of the unclear biological significance of negative effects, variability in effects and directions (positive or negative) may partly stem from variability in genetic background. We speculate that negative effects are caused by alleles that prevent a larval population from adopting a singular color. By the same token, there will be alleles that promote positive effects. This line of discussion is compatible with the previous findings (Figure 5 and Figure 6). Therefore, we conclude that the larval body colors were affected by both maternal and single-generation effects on the basis of various genetic backgrounds.

### 4.5. Possible Mechanisms of Maternal Effects

If crypsis is a function of body color in this species, the offspring generation of larvae have the “correct” information regarding the leaf color that they consume. In this sense, if the cryptic advantage of larval body color is important for survival, a single-generation effect may overwhelm the maternal effects. However, the present study suggests that both single-generation and maternal effects appear to be present. Considering the conditions for the evolution of the maternal effect according to Uller (2008) [43] (see Section 1), there may be a possibility that, in larvae, red pigment synthesis is more costly and requires a longer time to accumulate than green pigment synthesis. In contrast, red pigments or their related substances that are retained in the mother may be incorporated into the eggs at a relatively low cost. It is well known that a mother deposits biochemical materials in the egg cytoplasm, which is an important mechanism of maternal effects [41,42]. This line of argument is consistent with a decrease in the red larval frequency as the larvae grow in this species (Figure 3). In *A. puspa*, the red color was most evident at the late instars [38], in which case the larvae may execute a costly process of pigment synthesis without maternal effects. An alternative mechanism of the maternal effects observed in the present study is that mothers are able to extract chemical or visual information from the host plant that they consume at the larval stage, and such information is epigenetically transmitted to the offspring. In any case, because the dispersibility of this butterfly is low [45], there is a high possibility that an offspring generation may be born in the place where the parent generation was born. This means that the color of the host plant for the offspring generation will most likely be the same as the color of the host plant for the parent generation.

Based on our results (Figure 6), the maternal effect was evident only when mothers were red-fed and absent when they were green-fed. There are cases where paternal effects are also important [71,72,73]. However, because a paternal effect is considered much smaller than a maternal effect, in general, the paternal effect was considered negligible in comparison with the maternal effect in the present study. The paternal contributions should be clarified in the future.

Flavonoid pigments are present in the adults of many species of lycaenid butterflies and are sequestered from larval host plants [74,75,76,77]. Similar to other lycaenid butterflies, the pale grass blue butterfly sequesters flavonoids from the host plant leaves in the wing scales [78,79]. Creeping wood sorrel contains pigments (such as carotenoids and flavonoids) and other secondary metabolites [78,79,80,81,82]. There is a reasonable possibility that the maternal effects detected in the present study may be caused by the deposition of flavonoid precursors in eggs by mothers.

## 5. Conclusions

This study showed that the red form plant is suitable for the oviposition behavior of females and provides a larval diet that supports normal growth, although females prefer the green form plant. This study also showed that the larval body color is stage-dependent, providing basic information on the leaf and larval body colors in *Z. maha*. In addition to the stage-dependent effect, the plastic larval body colors of *Z. maha* are likely affected by the color of the leaves that larvae consume via single-generation effects and the color of the leaves that their mothers consume via maternal effects. The body color determination system of *Z. maha* appears to take advantage of these effects so as to enhance crypsis on two contrasting leaf colors.

The adaptive value of the green or red color of a given larva is not completely predictable at the egg stage, because a larva may be placed on either a green or red leaf on which its mother oviposited. This behavior of the mother may be affected by the availability of green or red leaves in the field. However, body color variation at the population level may be adaptive as a form of plastic crypsis in response to leaf color variation, and at the individual level, the larval body color may respond to the leaf color. It appears that both maternal and single-generation effects are maintained in this species in order to maintain responsiveness to fluctuating environmental conditions. The pale grass blue butterfly represents a good example of phenotypic plasticity using both maternal and single-generation information for survival in partially predictable environments.

## Data Availability

All the data generated or analyzed during this study are included in this published article and its Supplementary Materials.

## Supplementary Materials

The following supporting information can be downloaded at https://www.mdpi.com/article/10.3390/insects14020202/s1 as a single PDF file, which contains Table S1: Eclosion rate and survival rate after feeding on the red form or green form plant; Table S2: Single-individual tracking for body color scores (Line D-F_1_-red); Table S3: Single-individual tracking for body color scores (Line D-F_2_-red); Table S4: Single-individual tracking for body color scores (Line E-F_2_-red); Table S5: Single-individual tracking for body color scores (Line E-F_2_-green); Table S6: Eclosion rate and survival rate for Lines C, D, and E; Table S7: Eclosion rate and survival rate for Lines F, G, and H; Table S8: Larval body color scores; Table S9: Number of red/green larvae for the red larval frequency in Lines C, D, and E; Table S10: Number of red/green larvae for the red larval frequency in Lines F, G, and H.
