# Supplementary material for: The Plastic Larval Body Color of the Pale Grass Blue Butterfly Zizeeria maha (Lepidoptera: Lycaenidae) in Response to the Host Plant Color: The Maternal Effect on Crypsis
Source: Insects. 2023 Feb 17;14(2):202. doi: 10.3390/insects14020202 (PMC9966816; doi:10.3390/insects14020202)
Supplement: Supplementary file 1 [file insects-14-00202-s001.zip › insects-2157327-supplementary.pdf]

INSECTS

Supplementary Materials

The Plastic Larval Body Color of the Pale Grass Blue Butterfly *Zizeeria maha* (Lepidoptera: Lycaenidae) in Response to the Host Plant Color: The Maternal Effect on Crypsis

Ai Yoshida, Shintaro Yabu, Joji M. Otaki *

The BCPH Unit of Molecular Physiology, Department of Chemistry, Biology and Marine Science, Faculty of Science, University of the Ryukyus, Okinawa 903-0213, Japan

*Corresponding author at: University of the Ryukyus.
E-mail address: otaki@sci.u-ryukyu.ac.jp (Joji M. Otaki)

Tables S1 - S10

Table S1. Eclosion rate and survival rate after feeding on the red-form or green-form plant

Plant	Larva (*n*)	Pupa (*n*)	Eclosion (*n*)	Eclosion rate (%)	Survival rate (%)
Red form (Plant A)	20	20	19	95.00	95.00
Green form (Plant B-1, B-2)	99	98	97	98.98	97.98

Table S2. Single-individual tracking for body-color scores (Line D-F_1-red)

Individual number	17 September	19 September	20 September
1	3	3	0
2	3	3	2
3	3	2	0
4	3	2	0
5	3	2	0
6	3	2	0
7	3	2	2
8	3	2	2
9	2	3	2
10	2	3	3

Note: Dates indicate 2019.

Table S3. Single-individual tracking for body-color scores (Line D-F_2-red)

Individual number	16 October	19 October	20 October
11	3	2	0
12	3	3	2
13	3	2	2
14	3	3	0
15	3	2	2
16	3	2	2
17	2	1	1

Note: Dates indicate 2019.

Table S4. Single-individual tracking for body-color scores (Line E-F_2-red)

Individual number	4 November	6 or 7 November	8 November
18	3	3	2
19	2	1	1
20	2	2	2
21	2	2	2
22	2	1	1
23	2	1	1
24	2	1	1
25	1	1	1
26	1	1	1
27	1	1	1
28	1	1	1
29	1	1	1
30	0	1	1

Note: Dates indicate 2019.

Table S5. Single-individual tracking for body-color scores (Line E-F_2-green)

Individual number	1 November	4 November	6 November
31	3	2	1
32	3	3	1
33	3	3	2
34	3	3	2
35	3	1	1
36	3	3	2
37	2	1	0
38	2	2	0
39	2	0	0
40	2	2	0
41	2	1	1
42	2	1	0
43	2	1	1
44	2	2	1
45	1	0	0
46	1	1	0
47	1	1	0
48	1	1	0
49	1	1	0
50	1	0	0
51	1	1	1
52	1	1	1
53	1	1	1
54	1	1	1
55	1	2	1
56	1	2	0
57	1	1	1
58	1	1	1
59	0	0	0
60	0	0	0
61	0	0	0
62	0	0	0
63	0	0	0
64	0	0	0
65	0	0	0
66	0	0	0
67	0	0	0
68	0	0	0

Note: Dates indicate 2019.

Table S6. Eclosion rate and survival rate for Lines C, D, and E

Line	Generation	Plant	Larva (n)	Pupa (n)	Eclosion (n)	Eclosion rate(%)	Survival rate(%)
C	F_0	Red	20	20	19	95.00	95.00
C	F_0	Green	99	98	97	98.98	97.98
C	F_1	Red	320	300	293	97.67	91.56
C	F_2	Red	107	106	105	99.06	98.13
C	F_2	Green	171	169	169	100.00	98.83
C	F_3	Red	166	164	164	100.00	98.80
C	F_3	Green	98	96	94	97.92	95.92
D	F_0	Red	80	55	54	98.18	67.5
D	F_1	Red	97	92	91	98.91	93.81
D	F_1	Green	88	85	85	98.82	95.45
D	F_2	Red	237	87	76	87.36	-
D	F_2	Green	226	199	169	84.92	74.78
D	F_3	Red	35	20	6	30	17.14
D	F_3	Green	180	186	181	97.31	-
E	F_0	Red	37	55	54	98.19	-
E	F_1	Red	39	39	36	92.31	92.31
E	F_1	Green	126	112	108	95.54	84.92
E	F_2	Red	89	112	93	83.04	-
E	F_2	Green	155	194	177	91.24	-
E	F_3	Red	194	182	88	48.35	45.36
E	F_3	Green	147	131	64	48.85	43.53

Table S7. Eclosion rate and survival rate for Lines F, G, and H

Line	Generation	Plant	Larva (*n*)	Pupa (*n*)	Eclosion (*n*)	Eclosion rate(%)	Survival rate(%)
F	F_0	Green	237	222	221	99.55	93.25
F	F_1	Green	80	80	80	100.00	100.00
F	F_1	Red	182	164	156	95.12	85.71
F	F_2	Green	135	126	123	97.62	91.11
F	F_2	Red	64	59	57	96.61	89.06
F	F_3	Green	254	248	238	95.97	93.70
F	F_3	Red	85	65	56	86.15	65.88
G	F_0	Green	109	107	105	98.13	96.33
G	F_1	Green	360	334	325	97.31	90.28
G	F_1	Red	139	114	107	93.86	76.98
G	F_2	Green	144	131	129	98.47	85.58
G	F_2	Red	59	59	59	100.00	100.00
G	F_3	Green	377	370	368	99.46	97.61
G	F_3	Red	83	75	73	97.33	87.95
H	F_0	Green	241	235	229	97.95	95.02
H	F_1	Green	121	106	102	96.23	84.30
H	F_1	Red	86	75	69	92.00	71.88
H	F_2	Green	121	120	115	95.83	95.04
H	F_2	Red	36	30	30	100.00	83.33

Table S8. Larval body-color scores

Line	Generation	Plant	Score 0 (n)	Score 1 (n)	Score 2 (n)	Score 3 (n)	Number of larvae (n)
C	F_1	Red	91	181	130	51	453
C	F_2	Red	0	34	48	15	97
C	F_2	Green	57	51	52	7	167
C	F_3	Red	11	70	80	16	177
C	F_3	Green	6	37	42	14	99
D	F_0	Red	17	27	28	8	80
D	F_1	Red	18	36	28	15	97
D	F_1	Green	50	34	2	2	88
D	F_2	Red	50	95	69	23	237
D	F_2	Green	83	84	39	20	226
D	F_3	Red	11	16	7	1	35
D	F_3	Green	102	33	28	17	180
E	F_0	Red	6	22	6	3	37
E	F_1	Red	19	15	3	0	37
E	F_1	Green	51	51	16	8	126
E	F_2	Red	9	49	23	8	89
E	F_2	Green	45	68	27	15	155
E	F_3	Red	41	61	49	43	194
E	F_3	Green	41	58	25	23	147
F	F_0	Green	135	57	40	5	237
F	F_1	Green	19	21	40	0	80
F	F_1	Red	87	26	51	18	182
F	F_2	Green	60	40	29	6	135
F	F_2	Red	8	36	18	2	64
F	F_3	Green	116	119	19	0	254
F	F_3	Red	37	39	7	2	85
G	F_0	Green	52	27	27	3	109
G	F_1	Green	116	77	135	32	360
G	F_1	Red	12	61	61	5	139
G	F_2	Green	59	52	33	0	144
G	F_2	Red	28	23	8	0	59
G	F_3	Green	103	121	105	48	377
G	F_3	Red	11	40	16	16	83

H	F_0	Green	70	90	68	13	241
H	F_1	Green	38	34	40	9	121
H	F_1	Red	18	30	30	8	86
H	F_2	Green	32	31	48	10	121
H	F_2	Red	2	11	18	5	36

Table S9. Number of red/green larvae for the red larval frequency in Lines C, D, and E

Line	Plant	F_0	F_1	F_2	F_3
C	Red		362 / 91	97 / 0	166 / 11
D	Red	63 / 17	79 / 18	187 / 50	24 / 11
E	Red	31 / 6	18 / 19	80 / 9	153 / 41
C	Green			110 / 57	93 / 6
D	Green		38 / 50	143 / 83	78 / 102
E	Green		75 / 51	110 / 45	106 /41

Note: Numbers indicate (the number of red larvae) / (number of green larvae).

Table S10. Number of red/green larvae for the red larval frequency in Lines F, G, and H

Line	Plant	F_0	F_1	F_2	F_3
F	Red		95 / 87	56 / 8	48 / 37
G	Red		127 / 12	31 / 28	72 / 11
H	Red		68 / 18	34 / 2	
F	Green	102 /135	61 / 19	75 / 60	138 / 116
G	Green	57 / 52	244 / 116	85 / 59	274 / 103
H	Green	171 / 70	83 / 38	89 / 32	

Note: Numbers indicate (the number of red larvae) / (number of green larvae).
